# A real-time view of the TAR:Tat:P-TEFb complex at HIV-1 transcription sites

**DOI:** 10.1186/1742-4690-4-36

**Published:** 2007-05-30

**Authors:** Dorothée Molle, Paolo Maiuri, Stéphanie Boireau, Edouard Bertrand, Anna Knezevich, Alessandro Marcello, Eugenia Basyuk

**Affiliations:** 1 IGMM-CNRS UMR 5535, 1919, route de Mende, 34293 Montpellier, France; 2 Laboratory of Molecular Virology, ICGEB, Padriciano 99, 34012 Trieste, Italy

## Abstract

HIV-1 transcription is tightly regulated: silent in long-term latency and highly active in acutely-infected cells. Transcription is activated by the viral protein Tat, which recruits the elongation factor P-TEFb by binding the TAR sequence present in nascent HIV-1 RNAs. In this study, we analyzed the dynamic of the TAR:Tat:P-TEFb complex in living cells, by performing FRAP experiments at HIV-1 transcription sites. Our results indicate that a large fraction of Tat present at these sites is recruited by Cyclin T1. We found that in the presence of Tat, Cdk9 remained bound to nascent HIV-1 RNAs for 71s. In contrast, when transcription was activated by PMA/ionomycin, in the absence of Tat, Cdk9 turned-over rapidly and resided on the HIV-1 promoter for only 11s. Thus, the mechanism of trans-activation determines the residency time of P-TEFb at the HIV-1 gene, possibly explaining why Tat is such a potent transcriptional activator. In addition, we observed that Tat occupied HIV-1 transcription sites for 55s, suggesting that the TAR:Tat:P-TEFb complex dissociates from the polymerase following transcription initiation, and undergoes subsequent cycles of association/dissociation.

## Background

The human immunodeficiency virus type 1 (HIV-1) virus can have latent and acute phases. Latent viruses remain in infected organisms for a long time, and this prevents viral clearance by anti-retroviral agents. The control of HIV-1 latency is highly dependent upon transcriptional regulation: acutely-infected cells synthesize high levels of virus, while latently-infected cells transcribe little or no viral RNAs. HIV-1 transcription requires cellular co-factors, but it is highly activated by the viral protein Tat (for review, see [1,2]). In latent cells that do not express Tat, polymerases initiating at the HIV-1 promoter are poorly processive and do not transcribe the entire viral genome. However, extra-cellular signals can drive latent cells into acute phase by stimulating the HIV-1 promoter. Indeed, this induces the production of small amounts of Tat, which then initiates a positive feedback loop leading to full transcriptional activation [3].

Tat activates transcription by recruiting the active form of the positive transcription elongation factor P-TEFb to the HIV-1 promoter [4]. P-TEFb is composed of a complex between Cyclin T1 (CycT1) and the kinase Cdk9, and Tat directly binds CycT1 (see [5] for a review). Tat also binds TAR (trans-activation-responsive region), an RNA element present at the 5' end of all HIV-1 transcripts, and this induces the formation of a ternary complex on nascent RNAs, consisting of TAR, Tat, and P-TEFb. When incorporated in this complex, Cdk9 phosphorylates several components of the transcription machinery, including the C-terminal domain (CTD) of the large sub-unit of RNA polymerase II (RNAPII), and elongation factors DSIF and NELF [5]. This transforms RNAPII into a highly processive enzyme, which can transcribe the entire viral genome.

P-TEFb is not the only partner of Tat. In particular, Tat has also been shown to interact and recruit the histone acetyl-transferases p300 and PCAF, which can modify chromatin at the provirus integration site [1,2]. Moreover, Tat itself can be acetylated at Lysines 28 and 50, and these modifications have been shown to regulate its interactions with P-TEFb/TAR and PCAF [6-9].

While Tat and its various partners have been the subject of many studies, how these complexes behave in vivo is still a matter of debate. Indeed, several models currently exist. It has been proposed that P-TEFb dissociates from the HIV-1 gene following transcription initiation, while Tat and PCAF become transferred to the elongating polymerase [8]. In contrast, other models suggest that P-TEFb remains associated with Tat in the elongating complex [9,10]. To discriminate between these possibilities, we developed an assay to analyze the dynamic of the Tat:P-TEFb complex, directly in living cells and at HIV-1 transcription sites.

**Figure 1:**
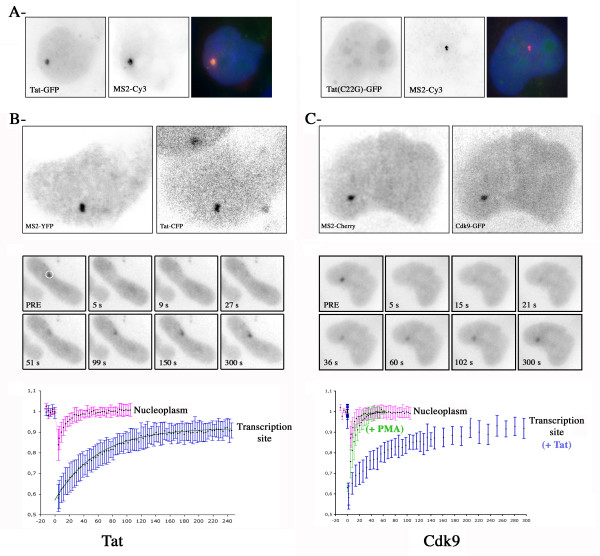
**Dynamic of Tat and Cdk9 at HIV-1 transcription sites. A-Accumulation of Tat, but not the C22G mutant, at HIV-1 transcription sites**. U2OS_HIV-1 cells were transfected with Tat-GFP or Tat(C22G)-GFP, and then induced 7h with PMA/ionomycin. Cells were then fixed and hybridized in situ with a Cy3-labelled oligo probe against the MS2 repeat. The HIV-1 transcription site corresponds to the focal accumulation labelled by the MS2 probe. Blue: dapi. Each field is 22 × 22 μm. **B-Dynamic of Tat at HIV-1 transcription sites. **U2OS_HIV-1 cells were transfected with vectors expressing Tat-CFP and MS2-YFP. Tat-CFP was then bleached, and recovery was analyzed by tracking transcription sites in 3D with a wide-field microscope. Upper panel: colocalization of Tat-CFP and MS2-YFP in living cells (30 × 25μm). Middle panels: image sequence from a FRAP experiment (time in second; each field is 30 × 25 μm). Graph: recovery curves in the nucleoplasm of transfected U2OS cells (pink), or at the HIV-1 transcription site (blue). The best fit is shown in green. **C-Dynamics of Cdk9 at HIV-1 transcription sites. **U2OS_HIV-1 cells were transfected with vectors expressing Cdk9-GFP and MS2-mCherry. Cdk9-GFP was then bleached, and recovery was analyzed by tracking transcription sites in 3D with a wide-field microscope. Upper panel: colocalization of Cdk9-GFP and MS2-mCherry in living cells (30 × 25 μm). Middle panels: image sequence from a FRAP experiment (time in second; each field is 30 × 25 μm). Graph: recovery curves in the nucleoplasm of transfected U2OS cells (pink), or at the HIV-1 transcription site. Blue: cells were transfected with Tat; Green: Tat was absent but cells were induced by PMA/ionomycin.

**Figure 2:**
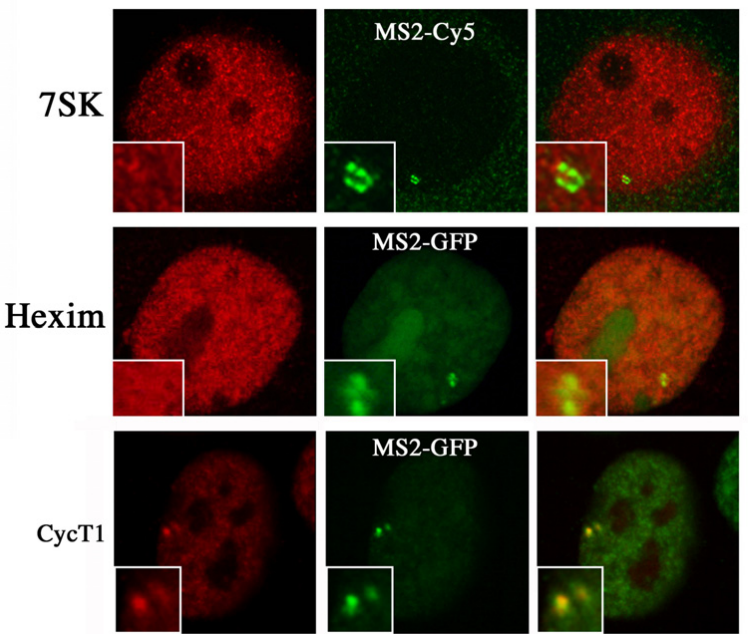
**Hexim and 7SK are not recruited at the HIV-1 transcription site**. U2OS_HIV-1 cells were transfected with vectors expressing Tat alone (upper panel), or Tat and MS2-GFP (middle and lower panels). 24h later, cells were fixed and processed. Upper panel: cells were hybridized in situ with fluorescent oligonucleotide probes detecting 7SK (red) or the MS2 repeat (green). Middle and lower panels, cells were labeled with antibodies against Hexim1 (red, middle panel), or cyclin T1 (red, lower panel).

Previous studies have shown that tagging RNAs with binding sites for the coat protein of phage MS2 allows its detection in living cells [11]. Thus, to visualize HIV-1 transcription sites, we tagged an HIV-1 vector with 24 MS2 binding sites [12]. This vector carried the elements required for RNA production: the 5' LTR that contained the TAR sequence, the major splice donor (SD1), the packaging signal Ψ, the RRE, the splice acceptor A7, and the 3' LTR that drives 3'-end formation. Stable clones expressing this reporter construct were generated in U2OS cells, and clones that showed robust trans-activation by Tat were further analyzed (clone U2OS_HIV-1, [12]). When expressed, the RNA was distributed homogenously in the cytoplasm and concentrated in a bright spot in the nucleoplasm. This spot corresponded to the transcription site as it was labeled with probes directed against the non-transcribed strand of the vector (data not shown, [12]). When the reporter was activated by Tat, several proteins accumulated at the HIV-1 transcription site, including Tat itself, and its cofactors Cyclin T1 and Cdk9 (Figure 1A and Figure 2). In contrast, this was not the case for either HEXIM-1 or 7SK, which together form a complex that inactivates P-TEFb ([13]; Figure 2). To test whether the accumulation of Tat at the HIV-1 transcription site depended on its interaction with Cyclin T1, we analyzed the localization of a point mutant of Tat, unable to bind Cyclin T1 (Tat C22G). U2OS_HIV-1 cells were transfected with fluorescent versions of Tat, and HIV-1 transcription was activated by treating cells with PMA/Ionomycin. Remarkably, while wild-type Tat accumulated at HIV-1 transcription sites, the C22G mutant did not (Figure 1A). Instead, it was homogeneously distributed in the nucleoplasm, even in the cells that contained high levels of nascent RNAs. This indicated that interaction with CycT1 was required to recruit Tat to HIV-1 transcription sites. In contrast, CycT1 and Cdk9 were recruited to the HIV-1 promoter irrespective of its mode of activation (Tat or PMA/Ionomycin; Figure 1C), consistent with the reported role of P-TEFb in transcriptional elongation [5].

To evaluate the dynamic properties of the TAR:Tat:P-TEFb complex, we performed experiments in live cells. We identified HIV-1 transcription sites with a yellow or red fluorescent variant of MS2, and performed photobleaching experiments (FRAP) on CFP and GFP-tagged versions of Tat and Cdk9 (see Additional file 1). When Tat or Cdk9 were bleached in the nucleoplasm of U2OS cells, the fluorescence recovered quickly, indicating that these proteins diffused rapidly through the nucleoplasm (Figure 1B and 1C). We then bleached Tat and Cdk9 at HIV-1 transcription sites, to analyze the dynamic properties of the complexes formed on nascent RNAs. The turn-over of Tat was slow, as complete fluorescence recovery took nearly three minutes (Figure 1B and Additional file 2). In the case of Cdk9, we observed two contrasting situations depending on the mode of activation of the HIV-1 promoter (Figure 1C). In the presence of Tat, Cdk9 recovery was also slow and took several minutes to go to completion (see Additional file 3). In contrast, when HIV-1 transcription was activated by PMA/ionomycin, Cdk9 was highly dynamic, and recovery was complete within seconds.

To extract more information from the FRAP recovery curves, each of them was fitted with a diffusion/binding model [14]. The assumptions made were that the binding sites are uniform within the volume of the spot and that their number does not change in time at steady state. This allowed us to derive some kinetic parameters: diffusion coefficients (D), residency time (τ_b_), and delay between two binding events (τ_d_; Table 1). The diffusion coefficients calculated for Tat and Cdk9 were similar and small, although they differed substantially in mass, pointing to a complex containing both proteins. We found that Tat remained bound to nascent HIV-1 RNAs for 55 seconds, and diffused for 60 seconds between two binding events. Similarly, in the presence of Tat, Cdk9 resided for 71 seconds at HIV-1 transcription sites, but diffused for 142 seconds between two binding events. This situation changed dramatically when HIV-1 transcription was activated by PMA/ionomycin, in the absence of Tat. In this case, P-TEFb turn-over was rapid, as it remained bound to the HIV-1 transcription site for only 11s. Since PMA/ionomycin promotes transcription by activating NF-kB, which directly recruits P-TEFb [5], a major conclusion of this work is that the dynamic properties of P-TEFb depend on its mode of recruitment to the HIV-1 promoter. It is interesting to speculate that the higher stability of the Tat:TAR:P-TEFb complex may account for the stronger activation obtained with Tat. A similar case has been observed for the transcription factor HSF (heat shock factor) in Drosophila [15]. Indeed, while HSF bound its target gene whether it is activated or not, the dynamic properties of this interaction varied dramatically upon transcriptional activation. This raises the possibility that it is not only the binding of transcription factors to their target genes regulates transcription, but also the dynamic properties of these events.

**Table 1:** Kinetic parameters of the fitted FRAP curves.

	**Tat**	**Cdk9 (with Tat)**	**Cdk9 (with PMA/ionomycin)**
**Diffusion coefficient**	8 μm^2^/s	7 μm^2^/s	9 μm^2^/s
**τ**_b_	55 s	71 s	11 s
**τ**_d_	60 s	142 s	15 s

τ_b _corresponds to the residency time at the HIV-1 transcription site. τ_d _corresponds to the time that separates two binding events at the HIV-1 transcription site.

Our data show that Tat and P-TEFb remained bound to nascent RNAs for about a minute. If HIV-1 transcription proceeds with the previously described rate of 2 Kb/min, then elongation through our reporter RNA would last more than 2 minutes [16]. This raises the possibility that the TAR:Tat:P-TEFb complex could be dissociated from the polymerase before the gene is completely transcribed. Following dissociation, the fate of Tat and Cdk9 is likely to differ, as shown by their significant difference in τ_d _(Table 1). It is remarkable that Tat and Cdk9 have very similar dynamics. This supports the idea that they remain together in the elongating complex, rather then Tat being transferred to the polymerase while P-TEFb dissociating from it. Since chromatin immunoprecipitation data have shown that Tat and P-TEFb are present with elongating polymerases all along the gene [10], we suggest that Tat and P-TEFb could undergo constant association and dissociation cycles with TAR and the elongating polymerase.

Altogether, our data show that the TAR:Tat:P-TEFb complex is remodeled during HIV-1 transcription. This work opens an opportunity to study the kinetic properties of factors involved in HIV-1 transcription, and could also be extended to the analysis of the contribution of post-translational modifications to the dynamics of the Tat:P-TEFb complex.

## Abbreviations

HIV: human immunodeficiency virus

RNAPII: RNA polymerase II

CTD: C-terminal domain of RNAPII

FRAP: fluorescence recovery after photobleaching

## Financial competing interests

The author(s) declare that they have no competing interests.

## Authors' contributions

D. Molle, S. Boireau, E. Bertrand, and E. Basyuk performed the FRAP experiments. D. Molle and A. Marcello performed the localization in fixed cells. P. Maiuri did the fitting of the FRAP curves. A. Knezevich realized the DNA constructs. E. Basyuk wrote the first draft of the paper. A. Marcello, E. Bertrand, and E. Basyuk elaborated the final version. This work was conceived by E. Basyuk and A. Marcello. All authors read and approved the final manuscript.

## Supplementary Material

Additional file 1 Experimental procedures. Experimental procedures used in the study are described. Click here for file

Additional file 2 Recovery of Tat at HIV-1 transcription sites. GFP-Tat was bleached at HIV-1 transcription site and stacks of images were taken every 3 seconds during 3 minutes after the bleach to monitor the recovery. Click here for file

Additional file 3 Movie 2. Recovery of Cdk9 at HIV-1 transcription sites, in the presence of Tat. CDK9-GFP was bleached at HIV-1 transcription site and stacks of images were taken every 3 seconds during 3 minutes after the bleach to monitor the recovery. Click here for file
