# Supplementary material for: A real-time view of the TAR:Tat:P-TEFb complex at HIV-1 transcription sites
Source: Retrovirology. 2007 May 30;4:36. doi: 10.1186/1742-4690-4-36 (PMC1904240; doi:10.1186/1742-4690-4-36)
Supplement: Additional file 1 — Experimental procedures. Experimental procedures used in the study are described. [file 1742-4690-4-36-S1.rtf]

Experimental procedures.
Cells and plasmids
U2OS cells were cultivated at 37°C in DMEM containing 10% FCS. For live cell experiments, cells were plated on glass, transiently transfected with lipofectamin with vectors expressing florescent versions of Tat, Cdk9, and MS2, and analyzed 24 hours later at 37°C in a non-fluorescent media [1]. 
Plasmids expressing Tat-eGFP, Cdk9-GFP, and GFP-CycT1 have been described previously [1-3]. Tat(C22G) was a gift of M. Benkirane. GFP-tagged CDK9 was obtained by PCR. MS2-Cherry was created with the Gateway system (Invitrogen). The MS2 tagged HIV-1 reported was derived from the plasmid pEV731 [4], by cloning 24xMS2 repeats into the ClaI-XhoI sites. Detailed information is available upon request.

In situ hybridization and immunofluorescence
In situ hybridization and immuno-fluorescence was performed as previously described [1; 2]. Fluorescent images of fixed cells were captured either on a MetaLsm510 confocal microscope (100x, NA 1.4, Zeiss), or on a DMRA wide-field microscope (100x, NA 1.4, Leica), equipped with a coolsnapHQ (Roper scientific) and controlled by Metamorph (Universal Imaging). 
	 
FRAP 
A Nikon TE200 equipped for both confocal and wide-field imaging was used (100X, NA 1.45). It was equipped with a piezzo-motor to capture z-stacks. This allowed to track transcription sites in 3D and to correct for cell movements. Transcription sites were bleached with the confocal port, using a circular region of 2.5 micron of diameter (bleaching time: 1s). Recoveries were then recorded using the wide-field port, with an excitatory light of low intensity. Images were recorded with an EM-CCD camera (Cascade 512K, Roper Scientific). Stacks of 9 images 0.5 micron apart were collected every 3 to 5s (one stack took 0,5-1 second). For image analysis, fluorescence intensities were measured in a small parallelepiped (1x1x1.5 micron) placed at the most intense area of the transcription site. Fluorescence intensities were normalized as in [5].

Curve fitting
The recovery curves of 10-20 cells were averaged and fitted with the Sprague/McNally model [6]. The best-fitting curve to the experimental data was obtained by the least squares fitting method.

References.
1.	D Fusco, N Accornero, B Lavoie, S Shenoy, J Blanchard, R Singer, E Bertrand: Single mRNA molecules demonstrate probabilistic movement in living mammalian cells. Curr Biol. 2003, 13:161-7.
2.	A Marcello, A Ferrari, V Pellegrini, G Pegoraro, M Lusic, F Beltram, M Giacca: Recruitment of human cyclin T1 to nuclear bodies through direct interaction with the PML protein. Embo J 2003, 22:2156-2166.
3.	M de la Mata, CR Alonso, S Kadener, JP Fededa, M Blaustein, F Pelisch, P Cramer, D Bentley, AR Kornblihtt: A slow RNA polymerase II affects alternative splicing in vivo. Mol Cell 2003, 12:525-532.
4.	A Jordan, P Defechereux, E Verdin: The site of HIV-1 integration in the human genome determines basal transcriptional activity and response to Tat transactivation. Embo J 2001, 20:1726-1738.
5.	R Phair, T Misteli: High mobility of proteins in the mammalian cell nucleus. Nature 2000, 404:604-9.
6.	BL Sprague, RL Pego, DA Stavreva, JG McNally: Analysis of binding reactions by fluorescence recovery after photobleaching. Biophys J 2004, 86:3473-3495.
